# Modification of Bacterial Nanocellulose Using Nonthermal Plasma-Assisted Enzymatic Hydrolysis

**DOI:** 10.1021/acs.biomac.5c00397

**Published:** 2025-08-05

**Authors:** Mirva Sarafidou, Aleksander Forys, Marcin Godzierz, Anastasiia Kobyliukh, Barbara Trzebicka, Stergios Pispas, Apostolis Koutinas, Erminta Tsouko

**Affiliations:** 1 Department of Food Science and Human Nutrition, 68995 Agricultural University of Athens, Iera Odos 75, Athens 11855, Greece; 2 Centre of Polymer and Carbon Materials, 49559 Polish Academy of Sciences, M. Curie-Sklodowskiej 34, Zabrze 41-819, Poland; 3 Theoretical and Physical Chemistry Institute, National Hellenic Research Foundation, 48 Vassileos Constantinou Ave., Athens 11635, Greece; 4 Division of Genetics & Biotechnology, Department of Biology, National and Kapodistrian University of Athens, Zografou Campus, Athens 15784, Greece

## Abstract

This study explored the structural modification of bacterial cellulose (BC) through enzymatic hydrolysis using varying cellulase activities and substrate concentrations. Optimal hydrolysis conditions (50 U/g of BC; 20 g/L of BC) were established to balance recovery and homogeneity (yielding BNC1). Hydrolysis was further combined with nonthermal plasma by suspending BC into plasma-activated water (PAW) prior to hydrolysis (BNC2). In another approach, BC suspensions were pretreated using a plasma bubble reactor followed by hydrolysis (BNC3). BNC1 and BNC2 yields were similar (∼50%), suggesting that PAW regulated the pH during hydrolysis. BNC3 yield was significantly higher (78%) compared to BNC1, indicating that the generated radicals promoted chain modifications while minimizing glucose/cellobiose release. That dual approach led to BC defibrillation, as revealed by AFM/cryo-TEM. The reduction in melting temperature observed was correlated with a crystallinity drop. Dual enzymatic and plasma-assisted strategies offer novel-intriguing avenues to fine-tune the properties of cellulose nanocomposites for sustainable applications.

## Graphical abstract

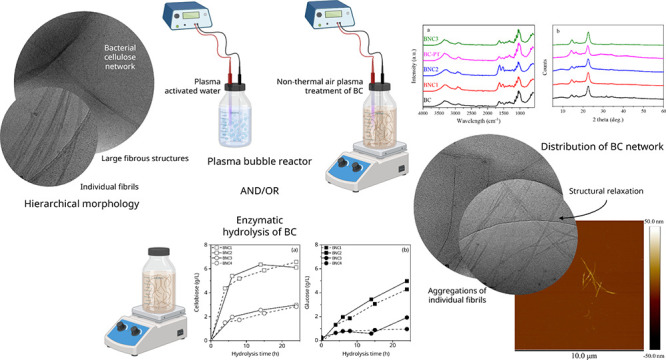

## Introduction

The growing demand for eco-friendly materials has intensified efforts to develop sustainable technologies to produce cellulose microfibers, nanofibers, and/or nanocrystals.^1^ Among potential sources, bacterial cellulose (BC), primarily produced by acetic acid bacteria, stands out as an alternative to plant cellulose, due to high purity and water retention capacity, superior thermomechanical properties, and inherent biocompatibility/biodegradability.^2,3^ Its distinctive 3D network makes BC a key material for sustainable, high-performance biobased products, with applications spanning biomedicine (e.g., drug delivery systems), food packaging, tissue engineering, biocomposites, and bioremediation.^4–6^

BC applications and processability can be improved through acid-assisted or enzymatic treatments that convert the material into nanostructured forms—such as microfibrils, nanofibrils, or nanocrystals—collectively referred to as bacterial cellulose nanostructures (BNCs). These strategies primarily target the amorphous regions of cellulose, disrupting the hydrogen bonding network and cleaving β-1,4-glycosidic bonds, which reduces fiber size.^7,8^ Consequently, the dense fibrillar network of BC is broken down into nanostructures with an increased surface area, higher crystallinity, and improved processability. BNC retains the cellulose I allomorph, predominantly in the *I*_α_ triclinic phase, which underlies its high crystallinity. This ordered molecular arrangement contributes to its exceptional mechanical strength, high aspect ratio, remarkable water-holding capacity, and excellent film-forming ability.^2^

Conventional strategies for cellulose nanostructure production often rely on strong acids, being associated with several drawbacks, including corrosiveness, toxic residues, large volumes of wastewater, low yields (losses throughout the process and material degradation), and complex processing steps.^9^ Consequently, these approaches face challenges in terms of sustainability and economic feasibility, underscoring the urgent need for greener, more efficient alternatives.^1,7,10^ Enzymatic hydrolysis has emerged as a promising method for structural modification of cellulose-based materials, providing controlled degradation coupled with safe and nontoxic end-products.^2,11^ Cellulases are complex enzymes, which act synergistically to break down cellulose into smaller fragments, including cello-oligosaccharides, cellobiose, or even glucose.^2,11^ The process begins with the random cleavage of the amorphous cellulose regions, eventually producing various hydrolysis products, while process efficiency depends on enzyme specificity, substrate characteristics, and reaction conditions. Crystalline regions are more resistant to enzymatic degradation due to strong hydrogen bonds, which are essential for enzyme binding on the hydrophobic crystal planes of cellulose.^12,13^ The enzyme’s capacity to preferentially target the amorphous structure can be strategically applied in the production of cellulosic microfibers, nanofibers, or nanocrystals with tailored properties.^14^ Guimarães et al.^11^ studied a multistage BC hydrolysis process by a commercial cellulase targeting the production of cellobiose through the adsorption of endoglucanases onto the BC and β-glucosidase removal by medium separation. Rovera et al.^2^ investigated the production of BC nanocrystals via enzymatic treatment, leading to different morphologies (fiber thinning) depending on the initial enzyme/BC ratio. Overall, despite being environmentally friendly, enzymatic approaches are often limited by slow reaction rates and insufficient control over the structure of the final product, often necessitating additional innovations to consistently produce uniform cellulose nanostructures.

Over the past few decades, nonthermal plasma (NTP) has emerged as a highly effective, sustainable, and versatile technology to modify properties of biomaterials due to low energy consumption and minimal chemical usage.^15–19^ Plasma is characterized as an electrically conducting medium, rich in reactive radicals, unbound positive and negative ions, quanta of electromagnetic radiation, and strong electric fields, being able to modify material surfaces and cleave chemical bonds.^1^ So far, plasma technology has been widely applied in areas such as biomass pretreatment,^20^ food preservation,^21^ polymer degradation,^22^ surface and water decontamination,^23^ textile modification,^24^ and chemical synthesis.^23^ One application of NTP into chemical synthesis is the production of plasma-activated water (PAW) using various plasma sources, such as dielectric barrier discharge (DBD).^23^ PAW properties are mainly connected with its strong acid environment, along with high redox potential.^23^ During NTP, reactive oxygen and nitrogen species (RONS) are formulated. Those species are mainly nitrates (NO_3_^–^), nitrites (NO_2_^–^), hydrogen peroxide (H_2_O_2_), and ozone (O_3_), classified as long-lived species, as well as hydroxyl radicals (•OH), nitric oxide (NO•), superoxide (O^2–^), peroxynitrate (OONO_2_^–^) and peroxynitrites (ONOO^–^) classified as short-lived species.^23,25^ The UV radiation is also responsible for the generation of •OH from the dissolved O_3_. RONS are generally produced at the interface between the gas and water or within the water, and their concentrations are related to the biochemical activity of the PAW.^23,26,27^ Application of plasma technology in chemical synthesis has also been reported for the production of nitric acid using a plasma bubble reactor via an electrolyte-regulation strategy as an eco-friendly alternative for acid synthesis.^28^

To date, the majority of NTP strategies applied on cellulose-based materials have primarily focused on surface modification,^29^ altering hydrophilicity,^16,18,30^ introducing antimicrobial functionality,^31^ or enhancing biocompatibility and adhesion,^17,32^ thereby improving the overall material quality rather than inducing structural transformation or depolymerization, which remains rather underexplored. Even in cases where NTP was employed for BC purification, such as the study of Leal Vieira Cubas et al.,^16^ the modifications remained confined to the surface, offering chemical-free purification but without significant disruption of the internal fibrous network. Likewise, other NTP-assisted cellulose processings related to plant biomass to reduce recalcitrance have been used to enhance subsequent fermentation or digestion, with limited investigation into bulk structural alterations or the facilitation of hydrolysis.^20,33^ So far, the dual strategy of plasma-generated reactive species and enzyme-driven specificity, in which plasma radicals initially weaken the cellulose structure, followed by targeted enzymatic hydrolysis, has received limited attention.

In this study, we address a critical gap by exploring a combined NTP–enzymatic strategy to enable controlled defibrillation and tailored production of BNCs. Initially, the effects of varying enzymatic activities and substrate concentrations on BC modification were examined. Building on this, a synergistic approach was developed by integrating NTP with enzymatic hydrolysis, either by substituting distilled water with PAW or by applying plasma pretreatment to BC, prior to hydrolysis, using a plasma bubble reactor. This approach leverages plasma-generated reactive species under mild aqueous conditions to potentially enhance the enzymatic activity and promote more efficient cellulose modification. To the best of our knowledge, this is the first systematic application of such a dual-mode strategy to microbial cellulose for nanostructure engineering. Importantly, this method eliminates the need for harsh chemicals, offering an environmentally friendly alternative for nanocellulose fabrication. The resulting BNCs were thoroughly characterized in terms of morphology, chemical composition, thermal behavior, and mechanical properties, supporting the sustainable and tunable design of cellulose-based nanomaterials.

## Experimental Section

### BC production

The bacterium *Komagataeibacter rhaeticus* UNIWA AAK2 (isolated from Kombucha beverage) was kindly provided by Dr. Maria Dimopoulou (Department of Wine, School of Food Science, University of West Attica). The preparation of the fermentation media, BC production, and purification were performed as described by Tsouko et al.^3^ with some modifications. Briefly, the fermentation culture (containing 20 g/L glucose, 5 g/L peptone, 5 g/L yeast extract, 2.7 g/L Na_2_HPO_4_, 1.15 g/L citric acid, pH adjusted to 6 using 5 M NaOH) was inoculated with 10% (w/w) of a preculture medium (same composition with the fermentation medium, inoculated for 24 h, under 100 rpm and using 250 mL Erlenmeyer flasks), while fermentation was conducted in static trays (1.5 L working volume), at 30 °C for 10 days. BC was removed from the culture broth, treated with 0.5 M NaOH (30 min boiling) to remove impurities, and then washed repeatedly with tap water until a neutral pH was monitored. Sequentially, BC was lyophilized (New Life Scientific, US), comminuted using a hammer mill (ECB mill, London) equipped with a sieve size of 1 mm, and stored in a desiccator until further use.

### Enzymatic Hydrolysis of BC

Water suspensions of lyophilized BC at specified concentrations were prepared in 100 mL Duran bottles (with a total volume of 40 mL). To facilitate even distribution and minimize potential enzyme aggregation or localized saturation, the BC suspensions were first prehomogenized^2^ using an IKA Ultra-Turrax T25 basic at 10,000 rpm for 4 min. The pH of the suspensions was properly adjusted by using 5 M HCl. Finally, the BC suspensions were autoclaved (Systec VX-150, SYSTEC, Germany) at 121 °C for 20 min and cooled. The cellulase preparation from *Trichoderma reesei* (Sigma Aldrich) was used as provided, with an activity of ≥700 U/g as specified by the manufacturer. Enzyme dosing was performed in U_cellulase_/g of BC based on this specification. No independent activity assay was conducted. For each treatment, a defined volume (μL) of the aqueous enzyme solution was added directly to the reaction system to achieve the desired enzyme loading. Following the addition of the enzyme solution, reactions were continuously stirred on a hot plate stirrer (Witeg Labortechnik GmbH, Germany) (55 °C, 300 rpm) throughout the hydrolysis period while different enzymatic activities (25, 50, and 100 g BC for 48 h) and BC concentrations (10, 20, and 30 g/L, for 24 h) were investigated. All the enzymatic hydrolysis experiments were conducted in triplicate.

The sampling was performed at intervals of 4–8 h to monitor the production of glucose and cellobiose, as well as to monitor the formation of BNCs. Finally, the mixtures were centrifuged (Sorvall LYNX 6000 Superspeed Centrifuge, Thermo Fisher, US) at 9000 rpm (at 4 °C for 20 min) to terminate the enzymatic reaction. The resulting cellulosic material was collected, further washed, and centrifuged (Sorvall LYNX 6000 Superspeed Centrifuge, Thermo Fisher, US) iteratively until a neutral pH was achieved. Subsequently, the resulting BNCs were lyophilized (New Life Scientific, US), ground to produce a homogenized material, and stored until further characterization.

### Setup and Operation of the Plasma Bubble Reactor and Strategy Evaluation

The plasma bubble reactor (Leap100, PlasmaLeap Technologies, Sydney, Australia) is presented on Figure 1, and it consisted of (a) a 1 L Duran bottle, (b) an alternating current (AC) power supply unit, (c) an air supply unit providing atmospheric air continuously, (d) a high-voltage electrode (HVE) and a ground electrode (GE), and (e) an agitator providing constant agitation (strategy 2) using a magnetic stirrer. Both electrodes were immersed either in water to produce PAW (strategy 1) or in a BC suspension (strategy 2). Water served simultaneously as the grounding medium and cooling agent for the discharge. The HVE was covered by a quartz tube powered by the AC unit, enabling two distinct plasma discharge modes: DBD along the reactor barrel and spark discharge within the gas bubbles at the gas–liquid interface. This configuration positions the plasma near the gas–liquid boundary, promoting the efficient transfer of reactive species into the liquid phase and producing UV emission around the microbubbles. All the experiments were operated at atmospheric pressure and ambient temperature.^20^

**Figure 1:**
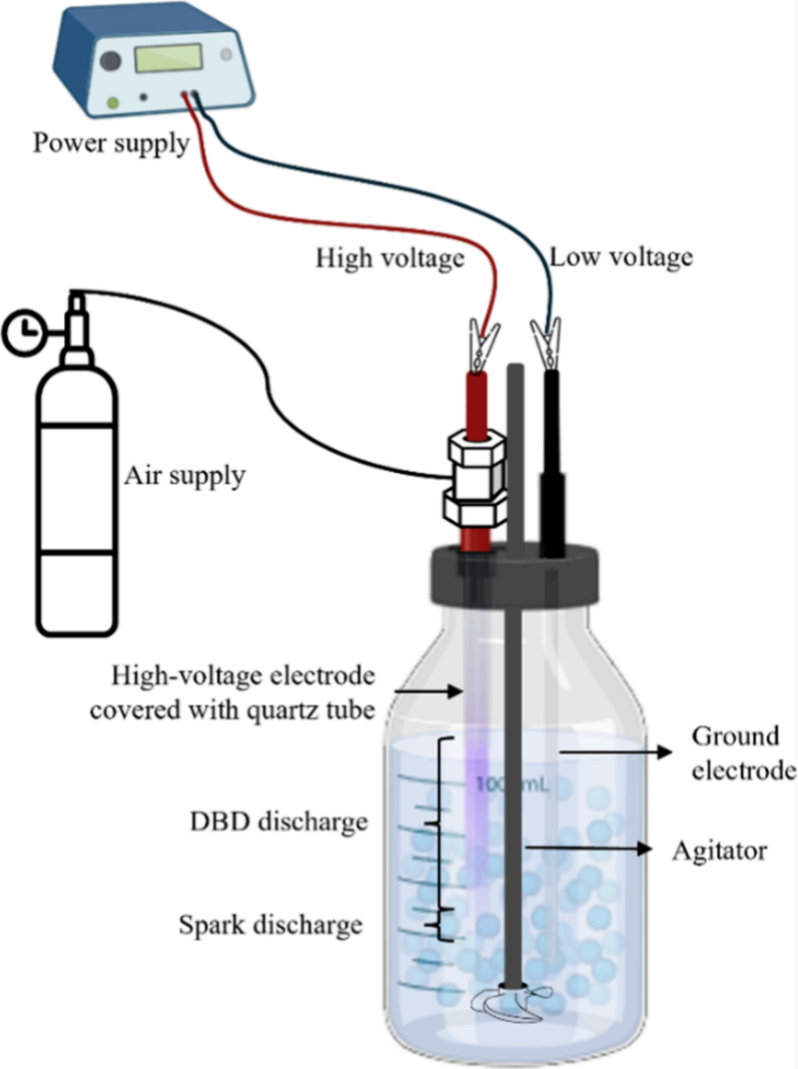
Schematic illustration of the plasma bubble reactor setup used in the present study.

#### Strategy 1: Production of PAW

The first strategy involved the substitution of distilled water during enzymatic hydrolysis with PAW. The production of PAW was achieved using a working volume of 800 mL of distilled water, 250 V voltage, 1.22 kHz frequency, 5.5% duty cycle, 3.3 vvm, and 40 min duration time. The PAW conductivity and pH were 1560 μS and 1.9, respectively. Enzymatic hydrolysis was performed utilizing 20 g/L BC and 50 U/g enzymatic activity, at initial pH (pH 5.0 ± 0.2) for 24 h, leading to the production of BNC2.

#### Strategy 2: Pretreatment of BC Suspension

In strategy 2, an aqueous BC suspension (20 g/L and 600 mL working volume) was pretreated using the plasma bubble reactor prior to the enzymatic catalysis. More specifically, the pretreatment of BC was conducted using distilled water, 200 V, 500 Hz frequency, 12.4% duty cycle, 2.0 vvm gas flow rate, and 60 min processing time. The subsequent enzymatic hydrolysis of the plasma-pretreated BC suspension (BC-PT) was carried out using a 50 U/g enzymatic activity at uncontrolled pH (pH 6.0 ± 0.2) for 24 h, leading to the production of BNC3. Untreated BC suspensions with a fixed pH at 6.0 ± 0.2 were used as control samples (BNC4) to investigate how deviations from the optimum (pH 5.0) affect the hydrolytic efficiency and structural modification of BC.

### Sugar Determination

Cellobiose and glucose concentrations were determined by high-performance liquid chromatography (HPLC) with a Rezex ROA-Organic acid H+ column and an RI detector (Shimadzu Corporation, Japan). The column temperature and the flow of the mobile phase (10 mM H_2_SO_4_ aqueous solution) were set at 65 °C and 0.6 mL/min, respectively.

BNCs yields were calculated according to Guimarães et al.^11^ as follows:

$$\mathrm{BNC}\;\mathrm{yield}\;(\%)=\frac{\mathrm{initial}\;\mathrm{BC}\;(g)-[\mathrm{cellobiose}\;(g)+\mathrm{glu}\cos e\;(g)]\times 0.9}{\mathrm{initial}\;\mathrm{BC}\;(g)}\times 100$$ 1

where 0.9 is the ratio between the molecular weight of cellulose monomer and glucose.

### BC and BNC Property Characterization

#### Atomic Force Microscopy (AFM)

AFM measurements were performed via a Dimension ICON AFM microscope equipped with a NanoScope V controller (BRUKER Corporation, Santa Barbara, CA, US) operating in the soft tapping mode in an air atmosphere with a standard 125 μm long, with flexural stiffness of 42 N/m of a single-crystal-doped silicon cantilever (Model PPP-NCH-10, NANOSENSORS). Images were obtained with a piezoelectric scanner with a nominal size of 85 × 85 μm. The micrographs were recorded with NanoScope Analysis 1.9 Software.

#### Cryogenic Transmission Electron Microscopy (Cryo-TEM)

Cryo-TEM images were obtained using a Tecnai F20 X TWIN microscope (FEI Company, Hillsboro, Oregon, USA) equipped with a field emission gun operating at an acceleration voltage of 200 kV. Images were recorded on a Gatan Rio 16 CMOS 4k camera (Gatan Inc., Pleasanton, California, USA) and processed with Gatan Microscopy Suite (GMS) software (Gatan Inc., Pleasanton, California, USA). Specimen preparation was done by vitrification of the aqueous solutions on grids with a holey carbon film (Quantifoil R 2/2; Quantifoil Micro Tools GmbH, Großlöbichau, Germany). Prior to use, the grids were activated for 15 s in oxygen plasma using a Femto plasma cleaner (Diener Electronic, Ebhausen, Germany). Cryo-samples were prepared by applying a droplet (3 μL) of the suspension to the grid, blotting with filter paper, and immediately freezing in liquid ethane using a fully automated blotting device, Vitrobot Mark IV (Thermo Fisher Scientific, Waltham, Massachusetts, USA). After preparation, the vitrified specimens were kept under liquid nitrogen until they were inserted into a cryo-TEM holder Gatan 626 (Gatan Inc., Pleasanton, USA) and analyzed in the TEM at −178 °C. The size of the fibers was measured using the ImageJ 1.47t software.

#### Attenuated Total Reflectance-Fourier Transform Infrared Spectroscopy (ATR-FTIR)

BC and BNCs were characterized by the ATR-FTIR technique. A Bruker Optik Fourier instrument (Tensor II, Equinox 55S) equipped with an ATR diamond accessory (SENS-IR) was used. A specified amount of lyophilized BC/BNC powders was placed on the diamond and stabilized by using a press. For BC/BNC samples, 50 scans were performed in the wavenumber range of 4500–750 cm^–1^ at a resolution of 4 cm^–1^. The analysis was performed at ambient temperature.

#### X-ray Diffraction Analysis (XRD)

The crystal structure of BC and BNCs was determined for powdered samples with the use of an XRD technique using a D8 Advance diffractometer (Bruker AXS, Karlsruhe, Germany). The Cu–Kα cathode (λ = 1.54 Å) operating at 40 kV voltage and 40 mA current was used as an X-ray source. The scan rate was 0.30°/min with a scanning step of 0.02° in the range of 5° to 80° 2Θ. Crystallinity index (CI) was calculated using the peak decomposition method. The crystallite size (CrS) was calculated using the Scherrer equation.^34^

#### Differential Scanning Calorimetry (DSC)

The melting temperatures (*T*_m_) and glass transition temperatures (*T*_g_) of BC and BNC samples were obtained from the first and second dynamic DSC run, respectively, in the range from 0 to 220 °C at 20 °C min^–1^. The DSC measurements were conducted in a dry nitrogen atmosphere (50 mL × min^–1^) for 5 mg powdered samples placed in nonhermetic aluminum pans, with the use of a differential scanning calorimeter (TA Instruments DSC Q2000).

### Statistical Analysis

The statistical analysis was carried out with STATGRAPHICS Centurion XVII, Version 17.2.00. The multifactor-ANOVA and Duncan multiple tests were performed to determine the statistically significant differences among the BNC yields with a 95% significance level.

## Results and Discussion

### Effect of Enzymatic Activity and Substrate Concentration on Hydrolysis Profile of BC

To reflect realistic and scalable process development, a complete cellulase system (commercial) from *Trichoderma reesei* was used rather than purified endoglucanases. This approach reflects industrial practices and supports our broader aim of establishing integrated and practical strategies for BC hydrolysis and modification using multienzyme preparations similar to those naturally secreted by fungi such as *Aspergillus awamori*. Τhe production of cellobiose and glucose during the enzymatic treatment of BC under various conditions, along with the corresponding BNC yields, is illustrated in Figures 2 and 3, respectively. The hydrolysis of cellulose with cellulases leads eventually to glucose production through the synergistic action of (a) endoglucanases, which randomly cleave cellulose chains (mainly amorphous regions) away from the chain ends to produce smaller cellulose fragments, (b) cellobiohydrolases the so-called exoglucanases, which act on the short crystalline regions on both chain ends (releasing cellobiose and low-molecular weight oligosaccharides), and (c) β-glucosidases, which specifically convert cellobiose into glucose.^2,35^ In the present study, the goal was to maintain minimal levels of cellobiose and glucose production while enhancing the modification and homogeneity of the BC slurry. BNC yields progressively decreased (Figure 3a) with increased enzyme activities due to the breakdown of BC into cellobiose and glucose (Figure 2a,b). The release trends of both cellobiose and glucose followed a similar pattern across all enzyme dosages. Cellobiose concentration peaked during the early stages (8–15 h), while it showed a consistent decrease with increasing enzyme activity over time, indicating the inversion of this dimmer into glucose. Correspondingly, glucose levels increased gradually until 48 h, consistent with sustained β-glucosidase action. Interestingly, cellobiose concentration (particularly after 24 and 48 h) showed a slight increase (*p* > 0.05) when increasing enzyme activity from 25 to 50 U/g, followed by a substantial decrease at 100 U/g (Figure 2a). At this enzyme dosage (50 U/g), exo- and endoglucanases likely release cellobiose at a rate that exceeds the conversion capacity of β-glucosidase, leading to its transient accumulation. At the highest enzyme loading (100 U/g), the system likely exhibits enhanced β-glucosidase activity, enabling more efficient conversion of cellobiose, as evidenced by significantly higher glucose concentrations and lower residual cellobiose levels.

**Figure 2:**
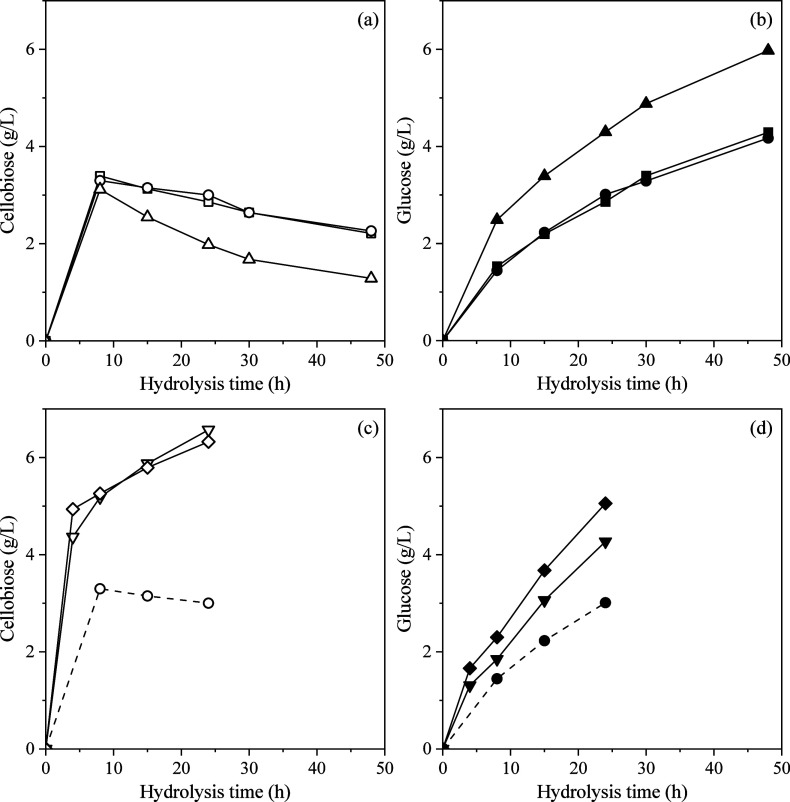
Cellobiose (a, c) and glucose (b, d) production during BC treatment. (a, b) Different enzymatic activities of 25 (square), 50 (circle), and 100 U/g (triangle) at 10 g/L initial BC concentration and pH of 5.0; (c, d) different BC concentrations of 10 (circle), 20 (inverted triangle), and 30 g/L (rhombus) at enzyme activity of 50 U/g and pH of 5.0. Dotted lines represent BC treatment under 10 g/L BC concentration, 50 U/g enzymatic activity, and pH of 5.0.

**Figure 3:**
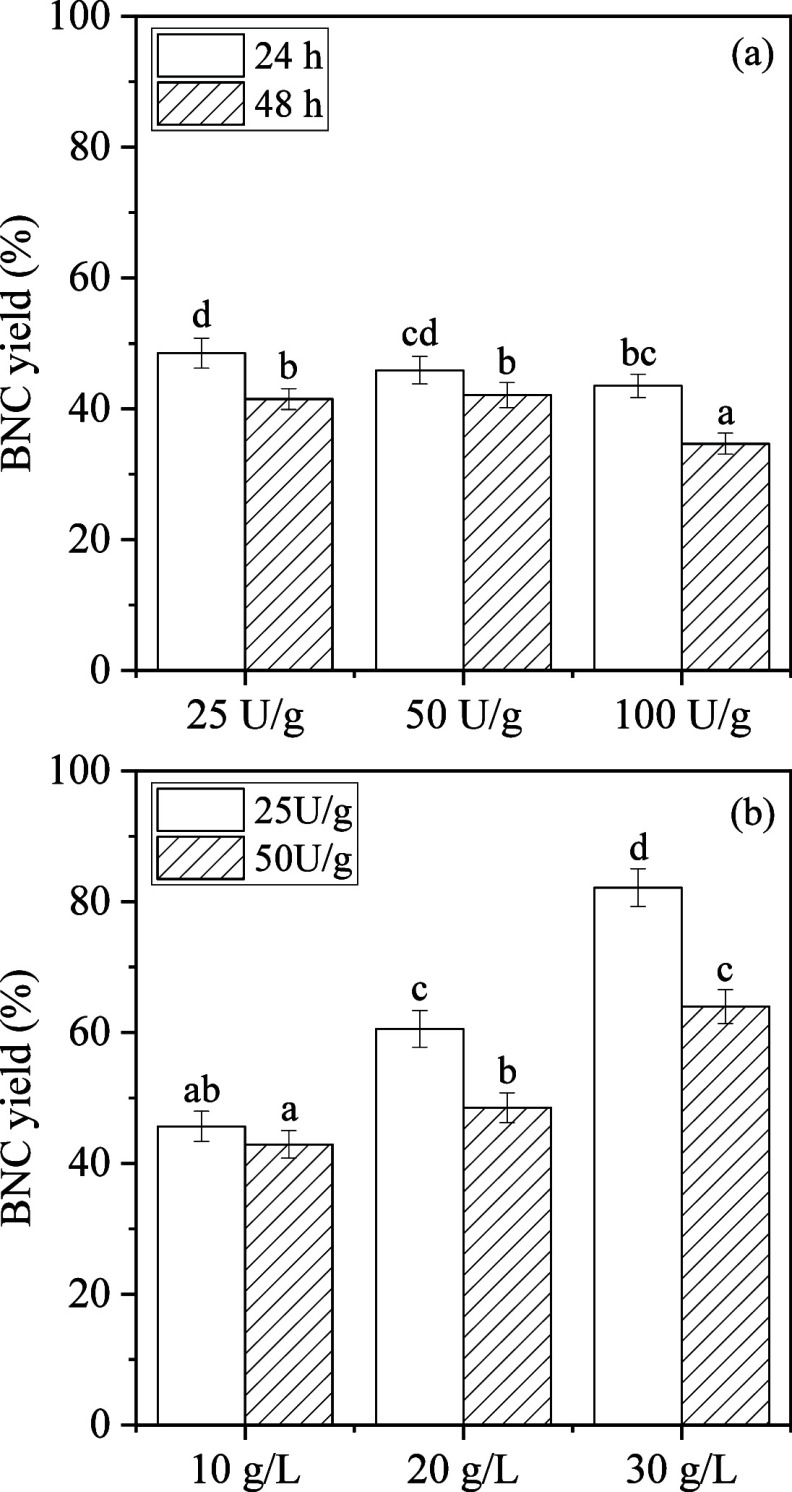
BNC yield after BC treatment applying different (a) enzymatic activities (25, 50, and 100 U/g) at 10 g/L BC, and pH 5.0; (b) initial BC concentrations (10, 20, and 30 g/L), and enzymatic activities of 25 and 50 U/g, at pH 5.0 after 24 h. Statistically significant differences (*p* < 0.05) are represented with different letters within the bars of the same figures.

BNC yields were not significantly affected (*p* > 0.05) when enzymatic activities of 25 and 50 U/g BC were applied (48.5 and 45.9%, respectively, at 24 h). The highest enzyme loading (100 U/g) resulted in the sharpest yield drop. Rationally, this was accompanied by the highest glucose production equal to 4.3 g/L at 24 h. Prolonging the hydrolysis process to 48 h resulted in lower BNC yields. Quite a lower yield (22.6%) was reported for nanocrystals prepared using commercial wheat microcrystalline cellulose after ultrasonic-assisted enzymatic hydrolysis for 120 h.^36^ In another study, BC produced by *Komagataeibacter xylinum* was enzymatically treated with yields of cellulose nanocrystals after 24 and 48 h, being 70 and 58%, respectively.^37^

Subsequently, the effect of varying initial BC concentrations was investigated at enzyme activity of 25 and 50 U/g after 24 h (Figure 3b). The dense and highly viscous environment created at elevated BC concentrations (20 and 30 g/L) posed significant challenges during experimental practice primarily due to improper mixing (creating zones of inadequate enzyme dispersion and localized substrate saturation), probably leading to reduced mass transfer and limited enzyme–substrate interactions.^38^ These limitations were particularly pronounced at the lower enzymatic dosage of 25 U/g (at both 20 and 30 g/L BC), and the highest BC concentration of 30 g/L (at both enzyme activities), where the limited enzyme’s ability to access the cellulose fibers was indicated based on optical observation. While BNC yields were notably higher in the case of 25 U/g at BC concentrations of 20 and 30 g/L, compared to 50 U/g enzymatic activity (Figure 3b), optical observation revealed that the BC slurries were nonuniform, with large, unhydrolyzed fragments persisting throughout the suspensions (Figure S1). This inconsistency suggested that 25 U/g enzyme activity was insufficient to fully overcome the physical barriers caused by high viscosity, resulting in incomplete and uneven processing of the biopolymer (Figure S2).^39^ These findings highlight the importance of the enzyme distribution in dense BC slurries. While not directly investigated in this study, this challenge underscores the need for improved delivery strategies in future work. Potential approaches include fed-batch enzyme supplementation, predilution of the BC slurry to reduce viscosity prior to enzyme addition, or the use of continuous high-shear mixing during hydrolysis. These strategies could enhance enzyme dispersion, improve contact with cellulose fibers, and ultimately optimize hydrolysis efficiency under high-solids conditions.

The release profile of cellobiose over time was very similar for both 20 and 30 g/L of BC at 50 U/g BC, which steadily increases (Figure 2c). More specifically, cellobiose concentration showed a significant rise after 4 h, with values of 4.4 g/L for 20 g/L BC and 4.9 g/L for 30 g/L BC. Thereafter, cellobiose production continued to increase, reaching up to 6.6 g/L for 20 g/L BC and 6.3 g/L for 30 g/L BC after 24 h. Similarly, glucose production steadily rose throughout the hydrolysis, reaching 4.3 g/L for 20 g/L BC and 5.1 g/L for 30 g/L BC at 24 h (Figure 2d). These results suggest that the differences in substrate concentration (20 g/L vs 30 g/L BC) may not be significant enough to alter the profile of cellobiose and glucose release in the given time frame. These findings align with the study of Rovera et al.,^2^ which shows that increasing BC concentrations resulted in less efficient hydrolysis compared to the enzyme-rich mixtures, highlighting the critical role of the enzyme-to-substrate ratio in the process. Additionally, the accumulation of cellobiose during the process likely contributed to further enzymatic inhibition, ultimately resulting in an undesirable outcome.^35^ Eventually, to obtain a balance between the product recovery (BNC yield = 51.2%) and homogeneity of the treated material, the enzyme activity of 50 U/g and initial BC concentration of 20 g/L were selected for further evaluation.

### Effect of Different NTP Strategies on BC Hydrolysis Profile

The interaction between NTP treatments coupled with enzymatic hydrolysis provides an intriguing avenue that may further improve the BNC yield and the final material performance. Herein, we investigated this combined approach with two different methodologies. The production of cellobiose and glucose, along with BNC yields, is presented in Figure 4, whereas the hypothesized fibril disintegration mechanism of BC by the different applied methodologies is presented in Figure 5. Initially, PAW was utilized as a substitute for hydrolysis water, allowing the enzymatic reaction to proceed under uncontrolled pH conditions, which stabilized at ∼5.0. Hydrolysis monitoring revealed similar trends in cellobiose and glucose production between the PAW-treated sample (BNC2) and BC that underwent enzymatic hydrolysis under the same pH using standard distilled water (control) (BNC1). Additionally, yields for BNC1 and BNC2 were nearly identical (*p* > 0.05), suggesting that the primary influence of PAW (pH_PAW_ = 1.9) was its ability to regulate the pH of the system without the need for external acidification. In the case of PAW, the long-lived species (H_2_O_2_, O_3_, NO_3_^–^, and NO_2_^–^) transfer bioreactivity to water, as short-lived species have half-lives up to a few seconds (Figure 5).^23,27^ The cleavage of C–C covalent bonds and hydrogen bonds presented in cellulose has been previously reported. Wen et al.^40^ confirm that the combination of O_3_, H_2_O_2_, and UV radiation reduces the degree of polymerization and increases the content of carboxyl groups. Wright et al.^41^ reported that O_3_ produced through plasma could damage the cellulose structure by selectively attacking C=C through ozonolysis. Huang et al.^42^ highlighted the ability of plasma treatment to reduce the inter- and intramolecular hydrogen bonds.

**Figure 4:**
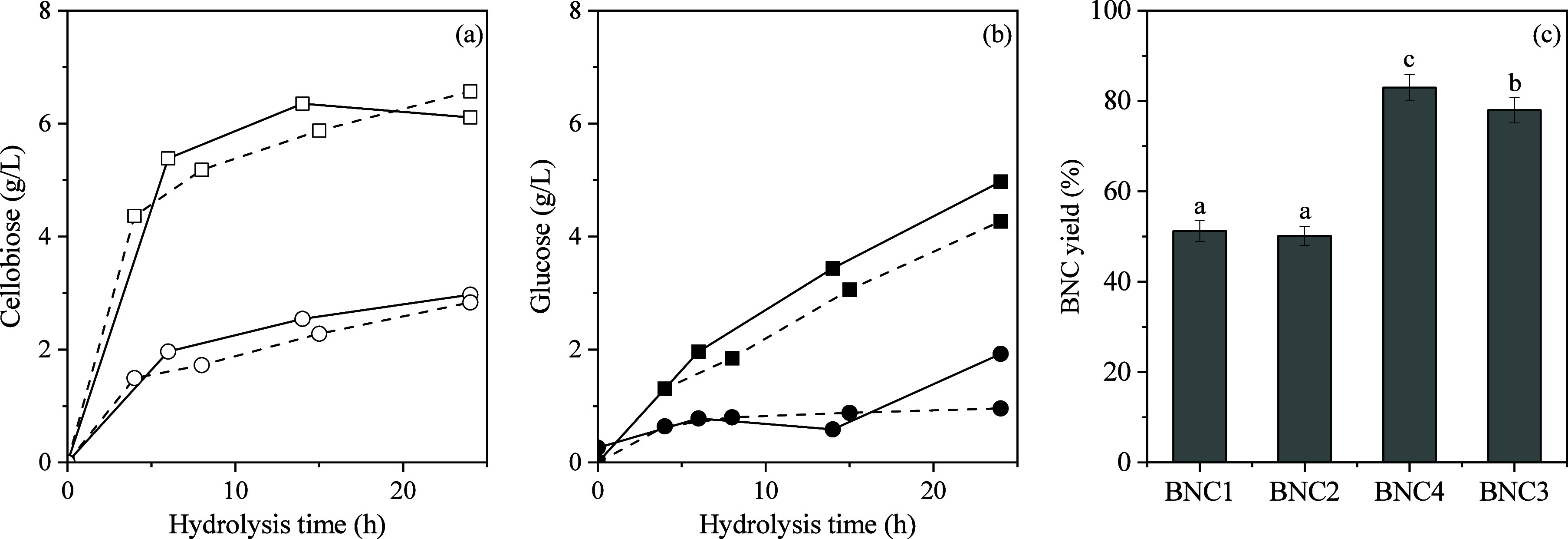
Cellobiose (a) and glucose (b) production along with the BNC yield (c) after different BC treatments (20 g/L BC, 50 U/g). Enzymatic hydrolysis at pH 5.0 using distilled water (BNC1, square-dotted line); enzymatic hydrolysis at pH 5.0 using plasma-activated water (BNC2, square-solid line); nonthermal plasma treatment followed by enzymatic hydrolysis at pH 6.0 (BNC3, circle-dotted line); enzymatic hydrolysis at pH 6.0 using distilled water (BNC4, circle-dotted line). Statistically significant differences (*p* < 0.05) are represented by different letters.

**Figure 5:**
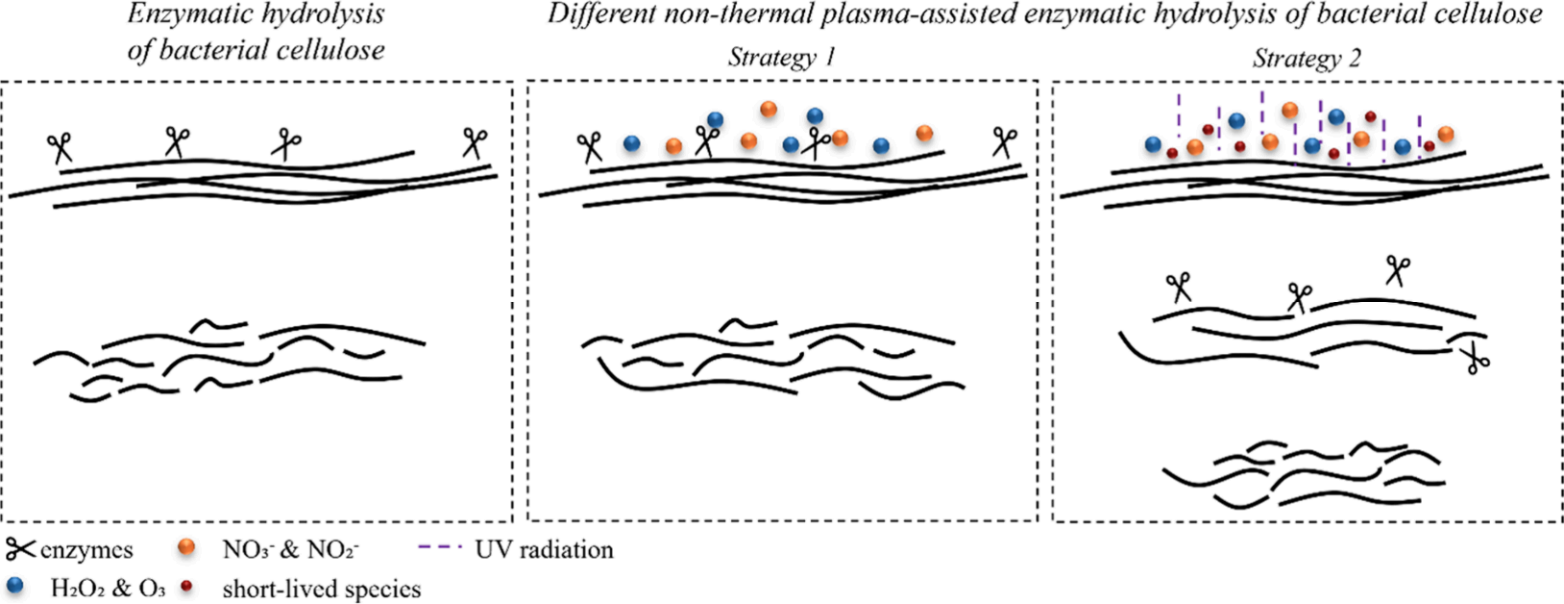
Fibril disintegration mechanism of BC by different applied methodologies.

In the second strategy, BC water suspensions were pretreated using a plasma reactor (BC-PT) prior to enzymatic hydrolysis, which was performed at a pH of 6.0 ± 0.2, leading eventually to the production of BNC3. As a reference, untreated BC suspensions with an adjusted pH value of 6.0 ± 0.2 were subjected to enzymatic hydrolysis to produce BNC4. In that case, it could be speculated that the particles interacting with BC are both short- and long-lived reactive species (Figure 5). The interaction of the short-lived species such as •OH and the UV photons with cellulose, as well as the cleavage of β-glucosidic bond and hydrogen bond, has been previously reported in the literature.^43,44^ BNC3 exhibited a significantly higher yield (78.0%) compared to BNC2, though it remained slightly lower compared to that of BNC4 (82.9%). The elevated BNC3 yield (and BNC4 yield), coupled with a low concentration of produced glucose (0.9–1.9 g/L) and cellobiose (∼2.9 g/L), initially suggested lower β-glucosidase and exoglucanase activities. BNC4 appeared less homogeneous and more viscous than BNC3 (Figure S1), indicating that in both cases, endoglucanases remained active, primarily modifying cellulose chains without excessive sugar release.^45^ However, BNC3 exhibited greater homogeneity due to plasma treatment.^41^ These observations align with findings by Hun and Catchmark,^46^ who investigated BC degradation using commercial cellulases in different buffer conditions for wound healing applications. Their study demonstrated that BC degradation occurs more slowly at pH 6.0 compared to pH 5.0 when using the same cellulase as in the present study. The reduced degradation rate at higher pH is attributed to a decreased cellulase adsorption rate, resulting in lower cellobiose and subsequently glucose concentrations.^47^ Additionally, the high BC concentration (20 g/L) and the viscosity of the slurry likely further hinder enzymatic access to reaction sites, leading to the slower degradation rate.^38^ Furthermore, the radicals generated during NTP treatment interact with glycosyl units on the cellulose surface, triggering the formation of additional reactive species that primarily cleave β-1,4-glycosidic bonds.^48^ The resulting free radicals may also remain trapped within the cellulose network after the process, potentially altering enzymatic accessibility, binding, and hydrolysis efficiency via the introduction of functional groups.^49^ In conclusion, the high pH during hydrolysis slows cellulose degradation into cellobiose and glucose, while the reactive species produced by NTP enhance the disruption of β-1,4-glycosidic bonds.

Further evaluation of plasma parameters, such as treatment duration, duty cycle, and gas composition, could potentially enhance its efficacy by changing the polarity and the morphology of BC.^17,18^ For instance, Rolim et al.^18^ reported that extending the pretreatment duration led to a decrease in the hydrophobicity of BC, highlighting the impact of plasma exposure on surface properties. Guided by these findings, the present study deliberately extended the plasma treatment duration to assess the effects of high exposure time on the BC structure and functionality. This strategy provided valuable insight into how prolonged plasma activation can modulate the fibrillar morphology. It also may serve as a foundation for future optimization studies, in which a wider range of plasma conditions will be systematically investigated. Moreover, the use of NTP in earlier studies to pretreat lignocellulosic substrates, targeting the reduction of the substrate recalcitrance and improvement of cellulose accessibility by altering holocellulose–lignin interactions, further supports the relevance of this approach to enhance enzymatic modification and improve BNC processability.^33^

### AFM and Cryo-TEM Investigations

AFM and cryo-TEM were employed to enable the visualization of BC and its hydrolyzed forms (BNC1, BNC2, and BNC3) in their near-native aqueous state (Figure 5). The depicted microstructures were carefully selected out of many images, as the observed characteristics may vary due to factors such as the specific observation area and structural differences within the same sample. AFM enabled a thorough structural assessment that captures both internal organization and surface features, crucial for applications in biomedicine, food packaging, and advanced biomaterials.^2,50^ The initial BC exhibited a complex hierarchical morphology of larger fibrous structures composed of smaller individual fibrils.^51^ The complex network of initial BC consisted of ribbons with a twisted morphology, which were measured at 6–7 μm in length and 90–100 nm in thickness (Figure 6a1 and Figure S3). This intricate arrangement was disrupted upon enzymatic hydrolysis, resulting in distinct structural modifications. AFM analysis revealed distinct morphological characteristics for all BNC samples, highlighting striations along the lengths of the cellulose ribbons. These striations likely result from the lateral aggregation of fibrils, a structural feature also observed in cellulose fibers described by Babi et al.^50^ BNC1 ribbons ranged from 0.6 to 4.3 μm in length, with a predominant fiber population being within 1.5–2.0 μm (Figure 6a2), reflecting the effective degradation of the BC network. The thickness of the primary fibers varied from 32 to 86 nm, indicating partial heterogeneity. BNC2 fibrils ranged from 0.7 to 5.0 μm in length, with a predominant population between 2.5 and 3.0 μm (Figure 6a3). The length and diameter of BC-PT fibrils ranged from 1.2 to 4.5 and 34 to 85 nm, respectively (Figure 5a4 and Figure S3). After enzymatic hydrolysis of the BC-PT sample, the BNC3 ribbons ranged from 1.1 to 4.8 μm in length (predominantly between 1.5 and 2.0 μm), with thickness varying from 46 to 85 nm, showing significant variability (Figure 6a5).

**Figure 6:**
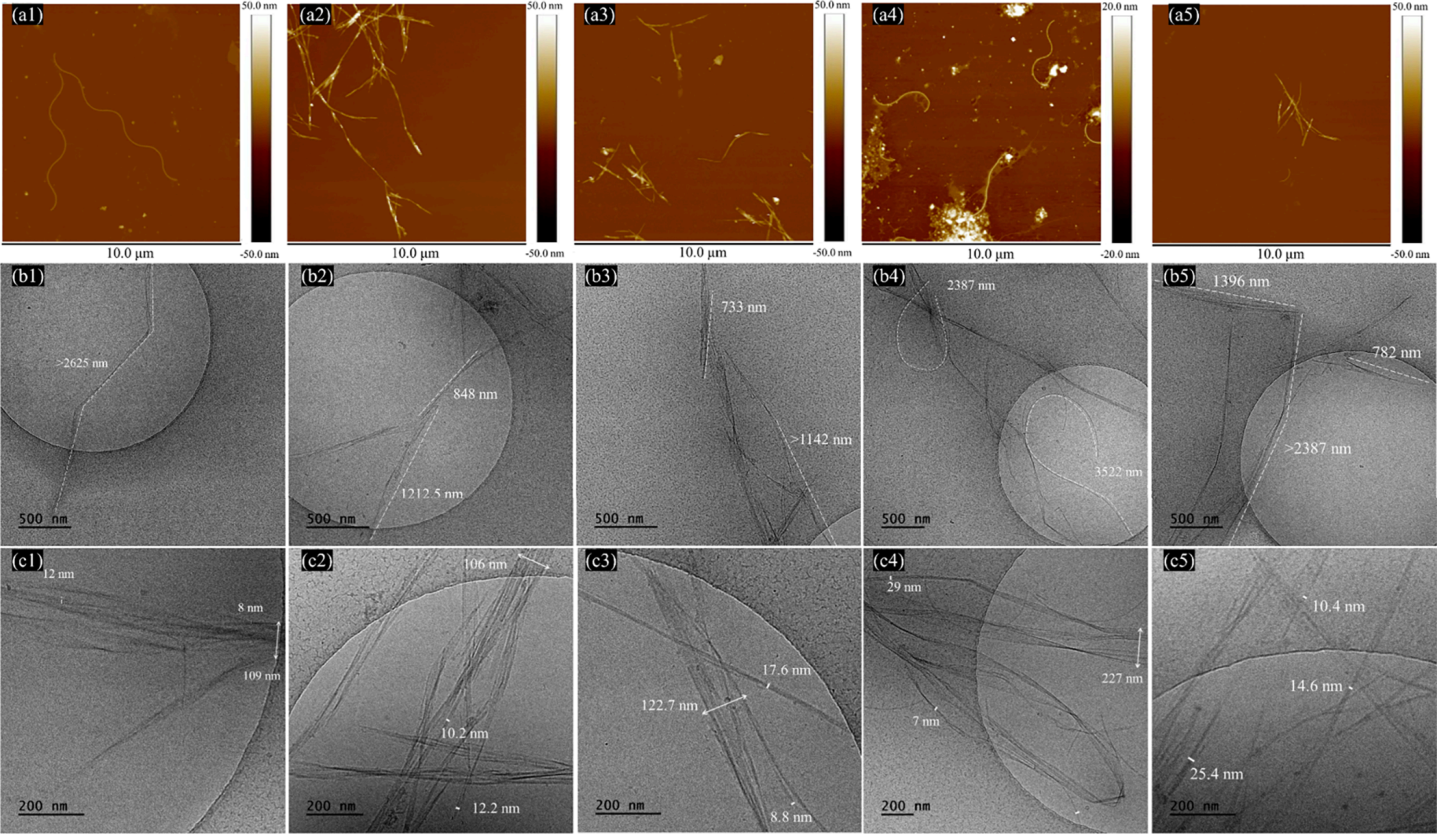
Structural morphologies of BC (1), BNC1 (2), BNC2 (3), BC-PT (4), and BNC3 (5) based on AFM height (a) and cryo-TEM (b, c) images.

BC and BNCs were further investigated using cryo-TEM (Figure 6b,c) to complement AFM, providing more detailed width measurements, due to higher lateral resolution.^50^ The overall fiber length reached 2.26 ± 1.14 μm (Figure 6b1), while the width displayed a multiscale organization. Primary fibers ranged from 50 to 75 nm in width (Figure 6c1), consisting of finer fibrils within 12–26 nm (Table 1), further enhancing the observations derived from the AFM images. All BNC samples exhibited similar morphologies, primarily consisting of heterogeneous, elongated ribbon-like structures formed by aggregations of individual fibrils. The thickness of individual fibrils ranged from 4 to 26 nm (Table 1), with length values higher than 0.5 μm, indicating the formation of nanofibrils.^52^ BNC1 fibrils displayed a thinner morphology (diameter = 7.4 ± 1.8 nm) and a reduced fiber length (0.86 ± 0.14 μm) compared to BC (Figure 6b2,6c2), reflecting the cellulase-induced disruption of its hierarchical structure (*p* < 0.05). When PAW was used instead of standard hydrolysis water (BNC2), the fiber length became lower without statistically significant differences (*p* > 0.05), with an average value of 0.81 ± 0.23 μm (Figure 6b3,6c3). The fibril widths demonstrated heterogeneity, ranging from 4.9 to 15.5 nm, and exhibited a significantly larger average diameter compared to that of BNC1 (*p* < 0.05) (Table 1). This suggests that PAW induced a mild structural effect on BC fibrils, possibly affecting their aggregation pattern and facilitating structural relaxation. BC-PT fibrils exhibited a slightly shorter average length (2.18 ± 0.95 μm) compared to untreated BC, though this difference was not statistically significant (*p* > 0.05). However, a significant reduction in fibril width was observed (*p* < 0.05), suggesting that the plasma treatment partially disrupted the hierarchical structure of the initial fibrils (Figure 6b4,6c4). For BNC3, fibril diameter and length were 7.4 ± 2.0 nm and 0.74 ± 0.17 μm, respectively (Figure 6b5,6c5), showing statistically significant differences compared to both BC and BC-PT (*p* < 0.05). The structural relaxation was clearly demonstrated, likely due to the combined effects of radical-induced modification and enzymatic cleavage.

**Table 1:** Diameter of BC and BNC Based on Cryo-TEM Analysis^a^

samples	diameter (nm)
BC	17.7 ± 3.7^c^
BNC1	7.4 ± 1.8^a^
BNC2	10.7 ± 3.2^b^
BC-PT	10.3 ± 5.0^b^
BNC3	7.4 ± 2.0^a^

a Different letters within the same column indicate statistically significant differences (*p* < 0.05).

The average aspect ratios (length-to-diameter) of BNCs exceeded 60, with BNC3 reaching values above 80, indicative of high mechanical integrity.^1,53^ The formation of thinner, elongated nanofibrils with high aspect ratios favors film formation with enhanced barrier properties. This is particularly advantageous for food packaging applications where maintaining a balance between mechanical integrity and a moisture barrier is essential. These morphological alterations align with findings from Rovera et al.^2^ who reported similar BC fibril dimensions and noted that increasing the enzyme-to-BC ratio transforms BC from an entangled fibril network into needle-like nanocrystals, either as individual structures or in aggregated forms. Additionally, all of the ribbon-like structures observed in the present study exhibited twisted morphologies, which could influence the mechanical and functional properties. Twisted fibrils have been associated with enhanced tensile strength, tunable optical properties, and altered interactions with water molecules, which may be relevant for applications in advanced biomaterials and functional coatings.^54^ These findings highlight the structural versatility of BNCs and suggest that both enzymatic and plasma-assisted treatments may offer tunability of the BC fibrillar architecture, facilitating the design of biopolymeric films with tailored tensile strength, flexibility, and moisture resistance.

### ATR-FTIR Characterization

The ATR-FTIR spectra of BC samples, untreated (BC) and pretreated with NTP (BC-PT) prior to enzymatic hydrolysis, as well as after enzymatic hydrolysis (BNC1, BNC2, and BNC3), are presented in Figure 7a. The broad absorbance bands observed at around 3400 and 3200 cm^–1^ are attributed to the O–H stretching vibrations (associated with both intermolecular and intramolecular hydrogen bonds) and −CH (aliphatic saturated CH_2_ and CH_2_OH), respectively.^55^ Notably, the −OH peak in BNC2 slightly shifted from 3338 to 3280 cm^–1^, suggesting enhanced intermolecular interactions which probably resulted from the formation of additional hydrogen bonds due to the incorporation of polar groups, likely as a result of PAW utilization.^17,56^ Moreover, samples subjected to enzymatic hydrolysis revealed a modest shift to higher wavenumbers in the case of the C–H stretching band (at 2918–2922 cm^–1^) compared to BC and BC-PT (at 2896 cm^–1^). The narrowing of the C–H stretching vibration of the cellulose in BNC1 and BNC2 correlates with the increased hydrolysis efficiency, which improves enzyme accessibility by increasing the surface area and generating more reducing and nonreducing ends along the cellulose chains due to cellulose disintegration.^41^ Considering the fingerprint region, the strong peaks at the areas 1640–1625 cm^–1^ and 1537–1525 cm^–1^ correspond to C=O stretching and N–H bending vibrations, respectively.^19^ These features may indicate the presence of amide groups, which have been previously reported in BC and are thought to arise from residual impurities (e.g., bacteria) in the cellulose network.^29,57^ Regarding BNC2, a shift to lower wavelengths was observed (from 1641 to 1626 and 1534 to 1525 cm^–1^), further supporting the observations of polar groups incorporation and oxidation increase in the presence of PAW.^1^ The bands at around 1428 and 1359 cm^–1^ are associated with C–H bending and C–OH in-plane bending vibrations, respectively,^16^ while the peak at 1315 cm^–1^ corresponds to the CH_2_ wagging vibration on the cellulose chain.^58^ The glycosidic bonds of the cellulose were identified by C–O–C symmetric and asymmetric stretching vibrations, which appear at approximately 1205 and 1160 cm^–1^, respectively.^57,59^ The peaks at around 1108 and 1031 cm^–1^ are characterized by the ring asymmetric valence and C–O stretching vibrations, respectively.^58^ Additionally, the peaks at 1055, 1076, and 929 cm^–1^ represent the C–O–C skeletal vibrations of the pyranose ring, C–H bending, and C–H out-of-plane bending, respectively.^19,60^ The region between 745 and 715 cm^–1^ exhibited two peaks characteristic of the *I*_α_ and *I*_β_ cellulose crystalline allomorphs, offering insight into the crystalline structure of the samples.^61^ The ratio of the peaks at ∼1430 and 898 cm^–1^ is also associated with the crystallinity of cellulose.^14,58^ Finally, the peak at ∼665 cm^–1^ is represented for the O–H out-of-plane bending vibrations.^58^ It is noteworthy that the spectra of the untreated BC and BC-PT samples show no significant differences, indicating that the structural integrity of the cellulose is largely maintained after NPT pretreatment. However, taking as a guideline the peak at 898 cm^–1^, a slight reduction of the peak at 1428 cm^–1^ occurred on BC-PT in contrast to BC, indicating crystallinity reductions. It has been reported that the cellulose crystalline phase is resistant to the argon plasma treatment (1% cellulose, 100 W, 30 min),^29^ whereas it is reduced in prolonged plasma treatment utilizing different gases.^42^

**Figure 7:**
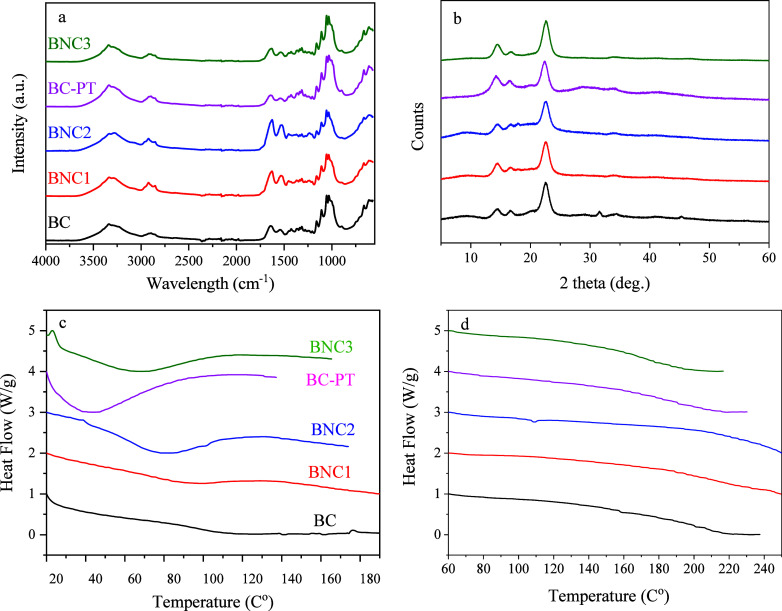
ATR-FTIR (a), XRD diffractograms (b), and DSC indicating the melting point (c) and the glass transition temperature (d) of BC, BNC1, BNC2, BC-PT, and BNC3.

### Effect of Different Treatments on Biopolymer Crystallinity

The XRD results for all samples are given in Table 2. All samples exhibited the typical structure of cellulose I, as indicated by three main diffraction peaks at 14.4, 16.7, and 22.6°. These peaks are commonly attributed to the (100), (010), and (110) crystallographic planes of triclinic structure *I*_α_ cellulose, respectively (Figure 7b).^62^ As the monoclinic structure *I*_β_ occurs, the peaks correspond respectively to (1–10), (110), and (200) crystallographic planes (Figure 7b).^14,59^ Cellulose *I*_α_ is the predominant allomorph of cellulose produced by acetic acid bacteria, e.g., *Komagataeibacter* species. Cellulose *I*_β_ is more characteristic of plants and tunicates, while some algae have been reported to contain both *I*_α_ and *I*_β_ types.^59^ Crystallinity significantly impacts the mechanical properties of the polymers. Higher crystallinity generally results in increased strength and stiffness since the ordered and tightly packed structures (crystalline regions) resist deformation under stress.^63^

**Table 2:** Crystallinity and Thermal Properties of BC and BNCs Based on the DSC Analysis[Table-fn t2fn2]

		CrS (nm)						
		peak position (2θ)						
samples	CrI (%)	14.4°	16.69°	22.6°	*T*_m_ (°C)	Δ*H*_m_ (J/g)	*T*_g_ (°C)	Δ*H*_g_ (J/g)
BC	76	6.1	7.9	5.9	117.5	21.3	202	0.3166
BNC1	68	6.0	5.5	5.6	90.5	10.1	207	0.4323
BNC2	70	5.7	5.3	5.3	72	73.7	176	0.3286
BC-PT	68	4.1	3.6	5.8	51	83.7	195	0.4114
BNC3	65	5.9	6.4	5.9	68	47.5	180	0.4608

a Crystallinity index (CrI); crystallite size (CrS); crystalline melting temperature (*T*_m_); melting enthalpy (Δ*H*_m_); glass transition temperature (*T*_g_); glass transition enthalpy (Δ*H*_g_).

BC presented the highest crystallinity index (CrI) (76%) among all examined samples, indicating a well-ordered structure, which is typical for untreated BC structures, being in alignment with findings from related studies.^14,59,64^ Different treatment strategies of BC, involving enzymatic hydrolysis alone or combined with NPT (under both strategies), led to a reduction of CrI within 8–14%. This suggests that enzymatic hydrolysis and additional treatments such as NTP may disrupt crystalline regions, leading to structural modifications. The reduction in CrI was also accompanied by a decrease in CrS, which is particularly evident for the (010) plane, which is associated with the lateral packing of cellulose chains. This is in line with findings reported by Arserim-Uçar et al.,^14^ where H_2_SO_4_- and HCl-treatment disrupted crystalline domains of BC, leading to a decrease in CrS (from 18.6 nm to 6.5–13.0 nm, regarding the (200) plane) and an increase in surface accessibility. This disruption indicates a weakening of intermolecular hydrogen bonding, which facilitates reactivity and improves processability for biopolymer applications.^14^ Notably, pretreatment of BC using a plasma bubble reactor resulted in both a lower CrI (68%) and a significantly reduced CrS (4.1 nm) of BC-PT compared to untreated BC (6.1 nm), suggesting that plasma treatment may have caused a partial amorphization of the cellulose structure.^34^ The most pronounced CrI reduction (65%) was observed in BNC3, indicating that the combined effect of plasma followed by enzymatic treatment led to the enhanced disruption of crystalline domains. The slight increase in CrS (5.9 nm) compared to that of PT-BC suggests that enzymatic hydrolysis, following plasma treatment, may selectively remove smaller disordered regions while partially stabilizing some of the remaining crystalline domains. Huang et al.^42^ highlighted the reduction of microcrystalline cellulose CrI from 83 to 59% after a 50 min pretreatment using a jet reactor that generates DBD plasma. This reduction was attributed to the generated radicals and ROS, whereas the presence of RNS has a secondary yet significant role as NO_2_ could degrade cellulose to glucose through oxidation.^41,65^ Similarly, Wright et al.^41^ utilized a plasma reactor that generates DBD, targeting the pretreatment of cellulose, leading to α-cellulose solubility rise from 17 to 70%, thus indicating crystallinity reduction. On the contrary, Cui et al.^36^ monitored a progressive increase in CrI (from 60.4 to 83.4%) during prolonged enzymatic hydrolysis (72–120 h) of microcrystalline cellulose. However, the hydrolysis time was significantly longer than that in our study (24 h), suggesting that the prolonged process may have led to the selective removal of amorphous regions, resulting in an apparent increase in crystallinity.

### Effect of Different Treatments on the Biopolymer Thermal Stability

Figure 7c,d shows the DSC thermograms of BC and BNCs. The heat-flow curves of BC showed an endothermic peak at 117.5 °C (Δ*H*_m_ = 21.34 J/g), which corresponds to the melting temperature (*T*_m_) of the crystalline region^63^ of the biopolymer. This relatively high value suggests the material’s good mechanical strength and resistance to thermal deformation, aligning with the high CrI of BC. At 202 °C, an exothermic peak was monitored, which is attributed to the glass transition temperature of the amorphous region (*T*_g_) of BC.^63^ Higher values of *T*_m_ = 136.7 °C (Δ*H*_m_= 87.7 J/g) and *T*_g_ = 350.7 °C (Δ*H*_g_= 86.7 J/g) were found by Vasconcelos et al.^64^ for BC produced from nata de coco.[Fig fig7]

BNCs exhibited different thermal behaviors compared to BC. More specifically, an endothermic peak occurred in lower *T*_m_ values (51–90.5 °C) while Δ*H*_m_ values ranged from 10.1 to 83.7 J/g. The most severe drop in *T*_m_ was observed for BNC2, BC-PT, and BNC3. Reactive species, oxidative radicals, and ions generated during NTP affect the thermal stability of cellulose, depending on the type of reactive gases and the treatment duration. Microcrystalline cellulose (obtained from cotton linters) after treatment with plasma (using Ar and Ar/N_2_ mixture) showed a slightly increased maximum decomposition temperature (*T*_max_) when the plasma duration was 30 min, while a duration time of 1 h led to decreased *T*_max_. ^1^

In this study, the reduction in *T*_m_ for BNCs correlates directly with the observed decrease in their CrI, suggesting a disruption of the crystalline regions due to enzymatic hydrolysis or plasma treatments. Additionally, the broader range of Δ*H*_m_ values for BNCs reflects the heterogeneity in their crystalline structure, likely due to varying degrees of hydrolysis and structural modification, as reported by AFM and cryo-TEM. These changes indicate a shift in the balance between the crystalline and amorphous regions in BNCs, with a relative increase in the amorphous content, facilitating the formation of nanofibrils. This can enhance flexibility and modify thermal properties, which may be advantageous for applications requiring tailored thermal responses or structural flexibility.^66^

The *T*_g_ of BNC1 was quite similar to that of BC, while when NPT and PAW were involved in the hydrolysis process, values decreased (176–195 °C). The higher surface area of BNCs (compared to BC) may have led to a higher exposed surface area to heat, thus resulting in decreased thermostability. The decomposition of BNCs at lower temperatures compared to BC might also be an indication of faster heat transfer within their matrix. Cellulose nanocrystals can act as effective channels for phonon transport, resulting in enhanced thermal conductivity.^67^ Nanocrystals of BC derived after strong acid hydrolysis have been reported to present an endothermic peak in the range of 140–195 °C with Δ*H*_m_ values ranging from 51 to 95 J/g.^64^ The presence of both endothermic and exothermic peaks in the BNCs highlights the coexistence of crystalline and amorphous regions. The *T*_g_ of BNCs (175–207 °C) suggests that the amorphous regions are quite thermally stable and exhibit relatively high rigidity. This characteristic contributes to the dimensional stability and resilience under thermal stress.

Considering the observed morphological and thermal transitions, several functional implications for the studied BNCs can be substantiated. The production of elongated nanofibrils with high aspect ratios, as indicated by AFM and cryo-TEM, substantially increases the available surface area for intermolecular interactions. This promotes the formation of a more cohesive and entangled network, which is expected to enhance film transparency, surface smoothness, and homogeneity—critical features in food packaging and biomedical materials.^68–70^ Moreover, the observed reductions in crystallinity and *T*_m_ in these BNCs indicate disruption of ordered domains and enhanced molecular mobility, consistent with a more flexible and less rigid polymer matrix.^71^ However, decreased crystallinity is also associated with increased water uptake and solubility, which can compromise the material’s moisture barrier performance.^57,69,72,73^ These opposing trends underscore the importance of balancing polymer dispersibility with mechanical and barrier functionality in reinforcement-related applications.

Importantly, the tunable morphology demonstrated in this study—through control over fibril dimensions and network density—enables the adjustment of the tensile strength, flexibility, and moisture resistance to meet specific application requirements. This structural adaptability enhances the practical potential of BNCs in biodegradable films, coatings, and functional composites, where mechanical integrity, barrier performance, and environmental compatibility must be simultaneously optimized.

## Conclusions

This study provided valuable insights into the enzymatic treatment of BC under varying enzyme activities and substrate concentrations with an emphasis on balancing glucose and cellobiose production, BNC yield, and material homogeneity. A substrate concentration of 20 g/L combined with an enzyme activity of 50 U/g provided an optimal balance between product recovery and structural uniformity. Further integration of the latter with NTP revealed that PAW effectively stabilized pH during hydrolysis without significant alterations in enzymatic efficiency. In contrast, pretreatment of BC using PBR prior to hydrolysis enhanced fibril relaxation and modulated enzymatic accessibility, leading to elevated BNC3 yield with minimal production of glucose and cellobiose. Structural characterization via AFM and cryo-TEM confirmed morphological transformations of BC after enzymatic and plasma-assisted treatments. XRD analysis indicated a reduction in crystallinity for all BNCs, suggesting a partial disruption of the ordered cellulose structure, which may have implications for its functional properties. Overall, these findings highlight the ability to control fibril architecture, crystallinity, thermal properties, and hydrolysis efficiency of BC, paving the way for advancements in biopolymer engineering, particularly in areas such as biomedical materials, nanocomposites, and functional coatings.

## Supplementary Material



## Notes

Images of BNC suspensions after 24 h of enzymatic hydrolysis; cellobiose and glucose production during BC treatment at enzyme activity of 25 U/g; structural morphologies of BC and BC-PT based on AFM and cryo-TEM images (PDF)

## Abbreviations

BC: bacterial cellulose
NTP: nonthermal plasma
PAW: plasma-activated water
DBD: dielectric barrier discharge
BNC: bacterial cellulose nanostructures
BC-PT: plasma-pretreated BC
HPLC: high-performance liquid chromatography
AFM: atomic force microscopy
Cryo-TEM: cryogenic transmission electron microscopy
ATR-FTIR: attenuated total reflectance-Fourier transform infrared spectroscopy
XRD: X-ray diffraction analysis
CI: crystallinity index
CrS: crystallite size
DSC: differential scanning calorimetry
Tm: melting temperature
Tg: glass transition temperatures
