# Supplementary material for: Modification of Bacterial Nanocellulose Using Nonthermal Plasma-Assisted Enzymatic Hydrolysis
Source: Biomacromolecules. 2025 Aug 5;26(9):5657–69. doi: 10.1021/acs.biomac.5c00397 (PMC12421519; doi:10.1021/acs.biomac.5c00397)
Supplement: Supplementary file 1 [file bm5c00397_si_001.pdf]

Supporting Information for

Modification of bacterial nanocellulose using non-thermal plasma-assisted enzymatic hydrolysis

Mirva Sarafidou[1], Aleksander Forys[2], Marcin Godzierz[2], Anastasiia Kobyliukh[2], Barbara Trzebicka[2], Stergios Pispas[3], Apostolis Koutinas[1], and Erminta Tsouko[4]**

[1] Department of Food Science and Human Nutrition, Agricultural University of Athens, Iera Odos 75, Athens 11855, Greece

[2] Centre of Polymer and Carbon Materials, Polish Academy of Sciences, M. Curie-Sklodowskiej 34, Zabrze 41-819, Poland

[3] Theoretical and Physical Chemistry Institute, National Hellenic Research Foundation, 48 Vassileos Constantinou Ave., 11635, Athens, Greece

[4] Division of Genetics & Biotechnology, Department of Biology, National and Kapodistrian University of Athens, Zografou Campus, 15784 Athens, Greece

*equally served as corresponding authors

Supporting information contains 3 pages including 2 Figures.

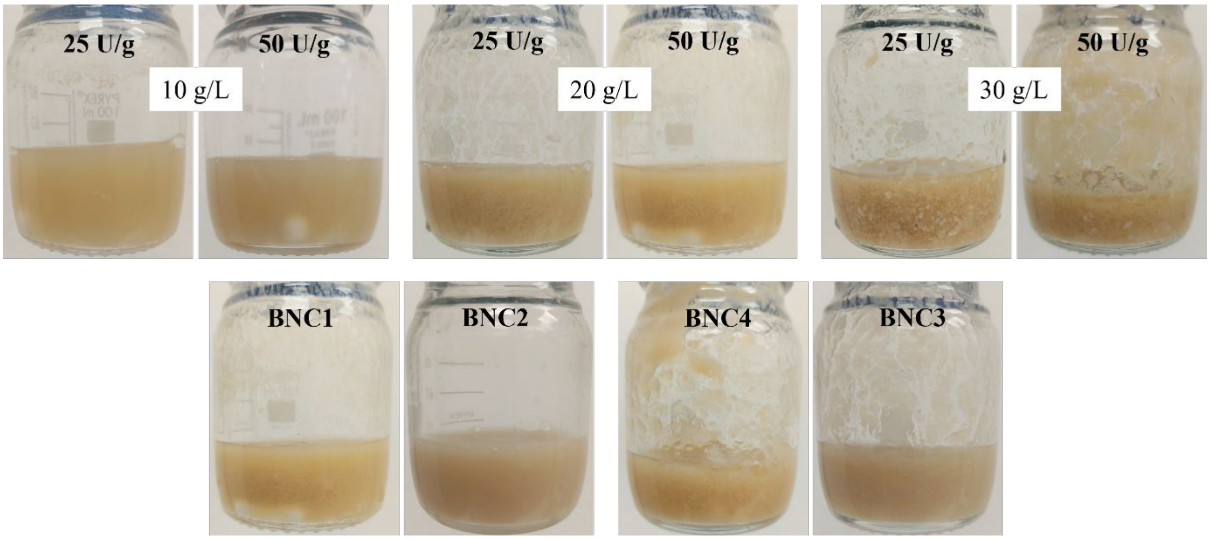

Figure S1. BNC suspensions after 24 h of enzymatic hydrolysis. The upper section is referred to three different initial BC concentrations (10, 20 and 30 g/L) and two enzymatic activities (25 and 50 U/g). The lower section is referred to BC treatment (20 g/L BC, 50 U/g) using NTP to produce BNC. BNC1, BC suspension in distilled water with fixed pH at 5.0 followed by hydrolysis; BNC2, BC suspension in PAW with pH 5.0 followed by hydrolysis; BNC4, BC suspension in distilled water with initial pH at 6.0 followed by hydrolysis; BNC3, hydrolysis of BC-PT suspension with initial pH at 6.0.

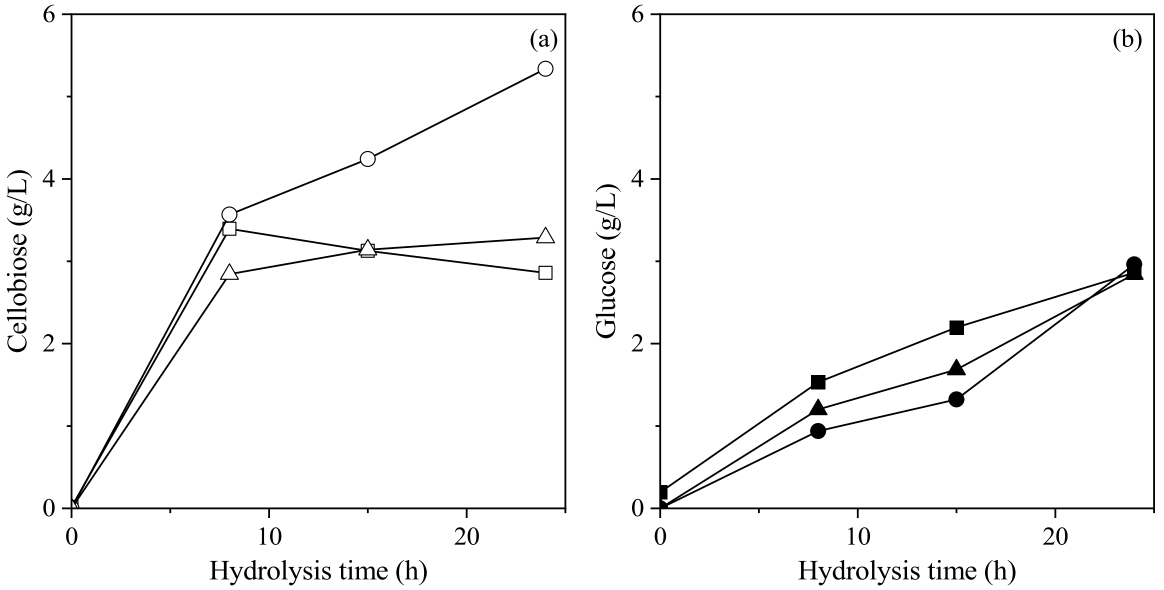

Figure S2. Cellobiose (a) and glucose (b) production during BC treatment using different BC concentrations of 10 g/L (square), 20 g/L (circle) and 30 g/L (triangle) at enzyme activity of 25 U/g and pH of 5.0.

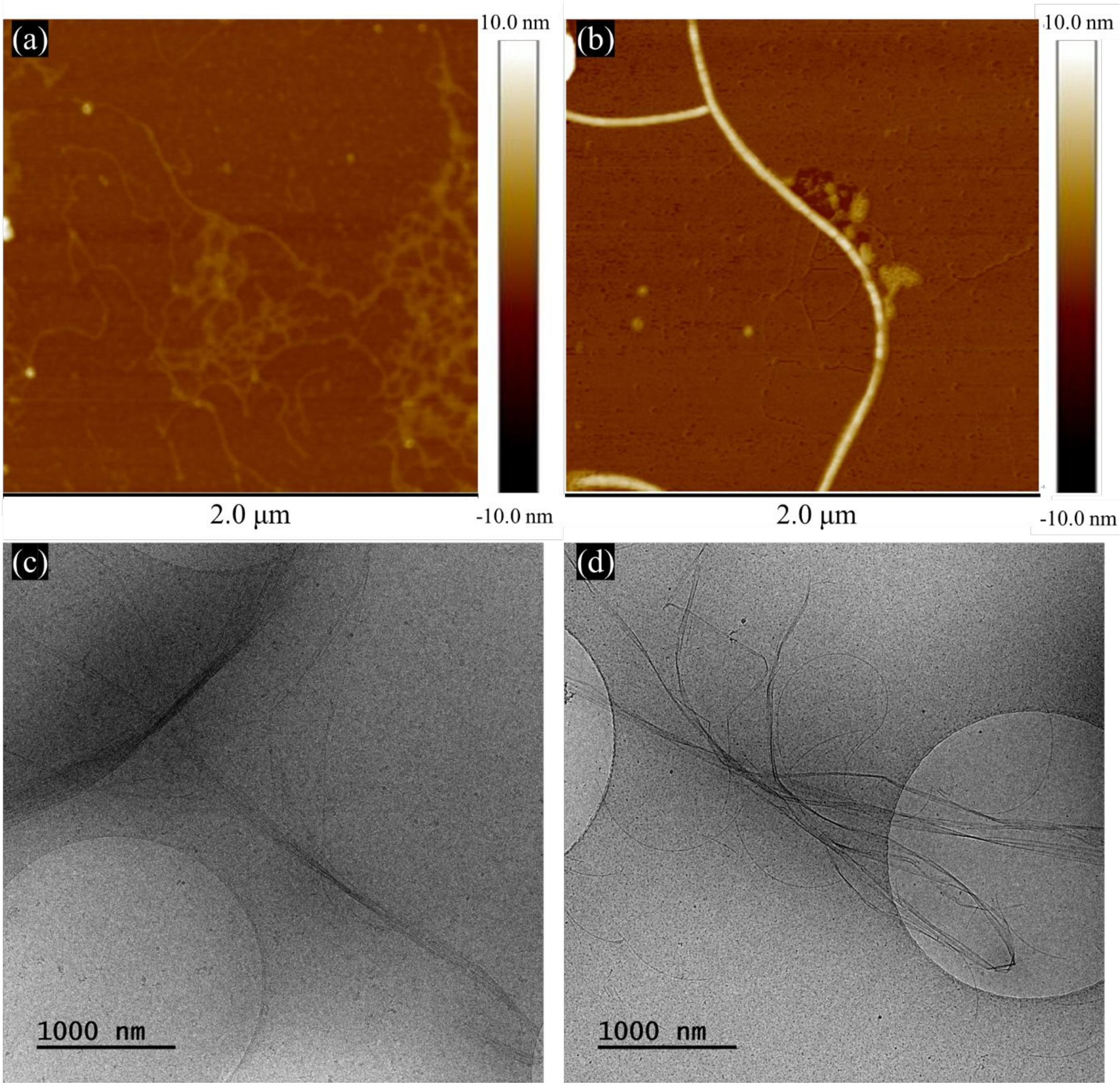

Figure S3. Structural morphologies of BC (a, c) and BC-PT (b, d) based on AFM height (a, b) and Cryo-TEM (c, d) images.
